# Individualized Therapy Guided by Drug Susceptibility Testing for Multidrug-Resistant Tuberculosis

**DOI:** 10.1093/ofid/ofag349

**Published:** 2026-06-18

**Authors:** Thomas Theo Brehm, Dagmar Schaub, Viola Dreyer, Niklas Köhler, Martin Kuhns, Florian P Maurer, Christoph Lange, Barbara Kalsdorf

**Affiliations:** Clinical Infectious Diseases, Research Center Borstel, Leibniz Lung Center, Borstel, Germany; German Center for Infection Research (DZIF), Hamburg-Lübeck-Borstel-Riems, Germany; Division of Infectious Diseases, I. Department of Medicine, University Medical Center Hamburg-Eppendorf, Hamburg, Germany; Clinical Infectious Diseases, Research Center Borstel, Leibniz Lung Center, Borstel, Germany; German Center for Infection Research (DZIF), Hamburg-Lübeck-Borstel-Riems, Germany; Molecular and Experimental Mycobacteriology, Research Center Borstel, Leibniz Lung Center, Borstel, Germany; Clinical Infectious Diseases, Research Center Borstel, Leibniz Lung Center, Borstel, Germany; German Center for Infection Research (DZIF), Hamburg-Lübeck-Borstel-Riems, Germany; Institute for Infection Research and Vaccine Development (IIRVD), Center for Internal Medicine, University Medical Center Hamburg-Eppendorf, Hamburg, Germany; Center for Clinical Studies, Research Center Borstel, Leibniz Lung Center, Borstel, Germany; Molecular and Experimental Mycobacteriology, Research Center Borstel, Leibniz Lung Center, Borstel, Germany; National and WHO Supranational Reference Laboratory for Mycobacteria, Research Center Borstel, Leibniz Lung Center, Borstel, Germany; Institute of Medical Microbiology, Virology and Hygiene, University Medical Center Hamburg-Eppendorf, Hamburg, Germany; Clinical Infectious Diseases, Research Center Borstel, Leibniz Lung Center, Borstel, Germany; German Center for Infection Research (DZIF), Hamburg-Lübeck-Borstel-Riems, Germany; Institute for Infection Research and Vaccine Development (IIRVD), Center for Internal Medicine, University Medical Center Hamburg-Eppendorf, Hamburg, Germany; Respiratory Medicine and International Health, University of Lübeck, Lübeck, Germany; Baylor College of Medicine, Global TB Program, Department of Pediatrics, Houston, TX, USA; Clinical Infectious Diseases, Research Center Borstel, Leibniz Lung Center, Borstel, Germany

**Keywords:** genotypic drug susceptibility testing, next-generation sequencing, phenotypic drug susceptibility testing, treatment outcomes, whole-genome sequencing

## Abstract

**Background:**

Drug-resistant tuberculosis remains a major health challenge, with treatment success for multidrug- or rifampicin-resistant (MDR/RR) disease substantially lower than for drug-susceptible tuberculosis. Access to accurate and timely drug susceptibility testing (DST) is essential for designing effective treatment regimens.

**Methods:**

We conducted a retrospective cohort study of adults with pulmonary tuberculosis treated at the Research Center Borstel in Germany between March 2019 and November 2021. Patients with culture-confirmed tuberculosis and either fully drug-susceptible disease or phenotypically confirmed MDR/RR tuberculosis were included. Comprehensive phenotypic DST (pDST) and molecular DST (mDST) by whole-genome sequencing (WGS) were performed. Treatment outcomes were assessed using the WHO 2021 definitions as the primary endpoint, with TBnet outcome definitions evaluated secondarily. Firth's penalized logistic regression identified predictors of cure.

**Results:**

Sixty-six patients were included: 43 with drug-susceptible tuberculosis and 23 with MDR/RR tuberculosis. Concordance between pDST and WGS-based mDST was high. In drug-susceptible disease, 13 (30%) were cured and 28 (65%) completed treatment, resulting in a WHO-defined success rate of 95%; 33 (77%) met TBnet cure criteria. Among MDR/RR patients, four (17%) were cured and 16 (70%) completed treatment, yielding a WHO-defined success rate of 87%; 19 (83%) were cured per TBnet criteria. No significant predictors of cure were identified.

**Conclusions:**

In this high-resource setting with access to comprehensive pDST and mDST, patients with MDR/RR tuberculosis achieved outcomes comparable to those with drug-susceptible disease. High concordance between phenotypic and molecular DST supports the reliability of mDST for guiding individualized treatment.

## Teaser

In a retrospective cohort of 66 adults with pulmonary tuberculosis treated at a German reference center, treatment outcomes were comparable between drug-susceptible and multidrug-/rifampicin-resistant tuberculosis, supported by high concordance between phenotypic and whole-genome sequencing–based molecular drug susceptibility testing enabling individualized therapy.

## Graphical Abstract

**Graphical Abstract:**
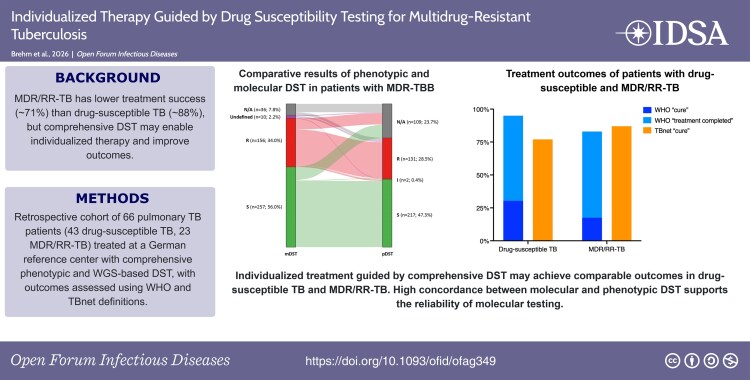
This graphical abstract is also available at Tidbit: https://tidbitapp.io/institutional-portal/open-forum-infectious-diseases/tidbits/individualized-therapy-guided-by-drug-susceptibility-testing-for-multidrug-resistant-tuberculosis/publish

Drug-resistant tuberculosis poses a significant global challenge, with an estimated 390 000 (95% uncertainty interval: 360 000–430 000) cases reported worldwide in 2024 [1]. While the treatment success rate for drug-susceptible tuberculosis is approximately 88%, it remains substantially lower at around 71% for multidrug-resistant or rifampicin-resistant (MDR/RR) tuberculosis [1]. For MDR/RR tuberculosis the number of effective drugs included in the treatment regimen has been shown to be directly correlated with clinical success, underscoring the importance of access to accurate drug susceptibility testing (DST) [2]. However, in many high-burden countries, the availability of both phenotypic DST (pDST) and molecular DST (mDST) remains limited, which compromises individual outcomes and threatens progress toward the End Tuberculosis Strategy goals [3]. We previously demonstrated that whole-genome sequencing (WGS)-based resistance prediction can effectively guide the design of personalized MDR/RR tuberculosis therapy regimens in a cohort of 70 patients treated at the Research Center Borstel in Germany, a national reference center for tuberculosis diagnosis and care [4]. Nevertheless, real-world studies directly comparing outcomes between patients with different resistance profiles in high-resource settings, where treatment is tailored by comprehensive pDST and mDST, remain scarce. Against this background, we aimed to compare treatment outcomes between patients with drug-susceptible tuberculosis and those with MDR/RR tuberculosis treated at the Research Center Borstel. Moreover, we assessed concordance between pDST and mDST and evaluated their implications for designing individualized treatment regimens.

## METHODS

### Study Population

We included all consecutive patients treated for pulmonary tuberculosis at the Research Center Borstel, Germany, between 20 March 2019, and 30 November 2021, who had positive *Mycobacterium tuberculosis* cultures on solid medium (ie, Löwenstein-Jensen and/or Stonebrink medium) or in the Mycobacteria Growth Indicator Tube (MGIT) system for pDST. Eligible patients had either fully susceptible strains (susceptible to rifampicin, isoniazid, pyrazinamide, and ethambutol) or phenotypically confirmed rifampicin resistance. Patients with rifampicin resistance detected only by Xpert MTB/Rif® (Cepheid, Sunnyvale, CA, USA) but not confirmed by pDST were excluded. Only patients who had received no more than seven days of antituberculosis treatment prior to admission to our center and who were not enrolled in a clinical trial were included. Patients with concurrent extrapulmonary tuberculosis manifestations were eligible provided that pulmonary tuberculosis was also present. All data were collected retrospectively through review of medical records and microbiology laboratory reports. The study was approved by the Ethics Committee of University of Lübeck under reference 2024-239.

### Phenotypic Drug Susceptibility Testing

pDST was performed in the liquid media culture system BACTEC MGIT 960 (Becton Dickinson, Sparks, MD, USA) according to the manufacturer's instructions. Standard critical concentrations were applied, with a critical proportion of 10% for pyrazinamide and 1% for all other drugs [5]. No clinical breakpoints were defined for cycloserine, para-aminosalicylic acid and meropenem. Cycloserine was tested using the indirect critical proportion method on solid medium (Löwenstein-Jensen medium), while para-aminosalicylic acid was tested in BACTEC MGIT 960. The breakpoints in use at the time of testing were applied [6].

### mDST Testing

The Xpert MTB/Rif® assay was carried out and evaluated in accordance with the manufacturer's instructions. In brief, 1 mL of raw sputum was mixed with 2 mL of sample reagent and incubated at room temperature for 15 minutes. Subsequently, 2 mL of this mixture was transferred to an Xpert MTB/Rif® assay cartridge and analyzed in a GeneXpert® instrument.

For MTBDRplus (Hain Lifescience, Nehren, Germany), DNA was extracted from sputum samples using the QIAamp DNA Mini Kit (Qiagen, Hilden, Germany) according to the manufacturer's instructions. For positive cultures, raw DNA was extracted by incubation at 95°C for 15 minutes and subsequent centrifugation at maximum speed for 5 minutes. Subsequently, 5 µL of the supernatant was used for further analysis. Amplification and hybridization procedures were performed according to the manufacturer's instructions.

For WGS, DNA was extracted from positive cultures using the cetyltrimethylammonium bromide-lysozyme method as previously described [7]. WGS was performed using a modified Nextera-based library preparation kit [8], and sequencing on an Illumina NextSeq 500/2000 platform (San Diego, CA, USA). Strains were sequenced paired-end with a target mean coverage of ≥50×. Raw reads were processed using the MTBseq pipeline v1.1.0 with default parameters [9]. Genome-based resistance prediction was performed by identifying mutations in genes known to be associated with drug resistance and comparing them with the WHO mutation catalog [10] and a previously published validated catalogue [9].

For comparative analyses, mDST results were categorized as resistant or susceptible based on the presence or absence of resistance-associated mutations. Mutations classified as uncharacterized or associated with undefined resistance interpretation in the available interpretation catalogs were recorded as a separate category and were not considered resistant or susceptible. Comparisons between mDST and phenotypic pDST were performed at the level of individual isolate–drug combinations. Analyses were conducted separately for drug-susceptible and MDR/RR tuberculosis to account for differences in diagnostic test coverage and resistance profiles between the two groups.

### Treatment Outcomes

Treatment outcomes were assessed according to the revised WHO 2021 definitions [11], which served as the primary endpoint for the statistical analyses. In addition, treatment outcomes were evaluated according to the TBnet criteria [12] as a secondary outcome measure. A successful outcome was defined as cure or treatment completion.

### Treatment Algorithms

A standardized algorithm [13] based on the WHO prioritization into group A, group B, and group C drugs [14] was applied using pDST results or mDST results as input to design personalized therapeutic regimens for each patient. For the purpose of treatment regimen construction, drugs with genomic results of unknown resistance significance were conservatively considered unsuitable for inclusion.

### Statistical Analyses

Agreement between pDST and mDST for patients with MDR/RR tuberculosis was assessed using Cohen's kappa coefficient. The analysis included drug–patient combinations with interpretable results for both methods (susceptible/resistant categories). Combinations classified as unknown mutation in mDST, not tested in pDST, or intermediate in pDST were excluded from the kappa analysis.

We also conducted univariate and multivariate analyses to identify factors associated with the WHO-defined treatment outcome cure. Firth's penalized logistic regression was applied to reduce bias related to the small sample size and the presence of rare outcome events. The following categorical variables were analyzed as potential predictors: tuberculosis drug resistance group (drug-susceptible vs MDR/RR), age (≤37 vs >37 years, corresponding to the cohort median age), sex, bilateral lung involvement, cavitary radiological pattern, extrapulmonary involvement, and body mass index (BMI ≥18.5 vs <18.5 kg/m^2^, corresponding to the WHO definition of underweight). Continuous variables were summarized using the median and interquartile range (IQR). Each predictor was first assessed using univariate Firth regression. All variables were then included simultaneously in a multivariate Firth logistic regression model. One patient with missing BMI data was excluded from the multivariable model using complete-case analysis. Odds ratios (ORs), 95% confidence intervals (CIs), and *P*-values were reported.

All statistical analyses were conducted using RStudio (version 2025.09.2+418) with R (version 4.2.2), employing Firth's penalized logistic regression as implemented in the *logistf* package, alongside *dplyr* and *broom*. Agreement between pDST and mDST was assessed using Cohen's kappa coefficient as implemented in the *irr* package.

Figures 2 and 4 were created using Microsoft Excel for Mac (version 16.104). Figure 3 was generated using RStudio (version 2025.09.2+418) with R (version 4.2.2), employing the *ggplot2* and *ggalluvial* packages for visualization.

## RESULTS

### Study Population

**Figure 1.**
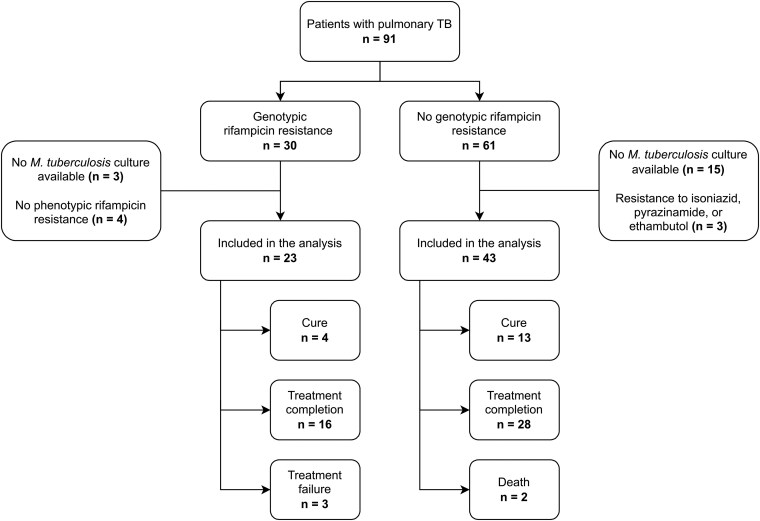
Flowchart of patient selection and treatment outcomes. Flowchart illustrating patient selection for inclusion in the study and treatment outcomes according to the revised WHO 2021 definitions. Abbreviation: TB, Tuberculosis.

Between 20 March 2019 and 30 November 2021, a total of 91 consecutive patients with pulmonary tuberculosis treated at the Research Center Borstel were evaluated (Figure 1). Among the 61 patients without genotypic rifampicin resistance detected by Xpert® MTB/Rif, 15 were excluded because no positive *M. tuberculosis* culture was available, and three were excluded due to monoresistance to isoniazid, pyrazinamide, or ethambutol. Of the 30 patients with genotypic rifampicin resistance detected by Xpert® MTB/Rif, 3 were excluded because no positive culture was available, and 4 were excluded because pDST did not confirm rifampicin resistance. All 23 remaining patients with confirmed rifampicin resistance also exhibited isoniazid resistance. Six patients had additional fluoroquinolone resistance consistent with pre-extensively drug-resistant (pre-XDR) tuberculosis, and 1 had additional bedaquiline resistance, meeting the criteria for extensively drug-resistant (XDR) tuberculosis [15].

In total, 23 patients with confirmed MDR/RR tuberculosis (including pre-XDR and XDR cases) and 43 patients with drug-susceptible tuberculosis were included in the final analysis.

### Baseline Characteristics

Patients with MDR/RR tuberculosis were younger (median age 30 years [IQR: 19.5–39.5] vs 50 years [IQR: 30–68]) and had a lower BMI (median 20.6 kg/m^2^ [IQR: 18.1–22.3] vs 22.3 kg/m^2^ [IQR: 19.8–23.9]) compared to patients with drug-susceptible tuberculosis (Table 1). The proportion of male patients was similar in both groups (65.2% vs 67.4%). Prior tuberculosis treatment was reported in 13.0% of MDR/RR tuberculosis patients and 11.6% of drug-susceptible tuberculosis patients. Smoking (current or past) was equally common in both groups (60.9% vs 60.5%). Intravenous drug use and HIV infection were reported exclusively in the MDR/RR tuberculosis group (8.7% and 13.0%, respectively). Alcohol abuse, diabetes mellitus, and chronic kidney disease occurred at similar frequencies across groups. Extrapulmonary tuberculosis involvement was present in 11.6% of drug-susceptible and 8.7% of MDR/RR tuberculosis patients. The median time to culture positivity was slightly longer in MDR/RR tuberculosis patients (10 days [IQR: 6.5–21.25] vs 8 days [IQR: 6.0–10.25]). Bilateral lung involvement was more common in drug-susceptible tuberculosis (76.7% vs 56.5%), whereas cavitary disease was more frequent in MDR/RR tuberculosis (56.5% vs 39.5%).

**Table 1.** Characteristics of the Study Population

	Drug-susceptible TB	MDR/RR-TB
*N*	43	23
Age in years, median (IQR)	50 (30–68)	30 (19.5–39.5)
Male sex, n (%)	29 (67.4)	15 (65.2)
BMI in kg/m², median (IQR)	22.3 (19.8–23.9)	20.6 (18.1–22.3)
Previous TB, n (%)	5 (11.6)	3 (13.0)
Smoking, n (%)	26 (60.5)	14 (60.9)
IVDA, n (%)	0	2 (8.7)
Alcohol abuse, n (%)	7 (16.3)	3 (13.0)
HIV, n (%)	0	1 (4.3)
Diabetes mellitus, n (%)	3 (7.0)	1 (4.3)
Chronic kidney disease, n (%)	3 (7.0)	0
Extrapulmonary involvement (%)	5 (11.6)	2 (8.7)
TTCP in days at treatment start, median (IQR)^a^	8 (6.0–10.25)	10 (6.5–21.25)
Bilateral disease, n (%)	33 (76.7)	13 (56.5)
Cavitary disease, n (%)	17 (39.5)	13 (56.5)

Abbreviations: BMI, body mass index; DS, drug-susceptible; HIV, human immunodeficiency virus; IQR, interquartile range; IVDA, intravenous drug-abuse; MDR/RR, multidrug-resistant or rifampicin-resistant; TB, tuberculosis; TTCP, time to culture positivity.

^a^Thirty-six patients with DS-TB and 18 patients with MDR-TB had a positive culture from sputum (and thus a TTCP-value) at treatment start.

### Treatment Outcomes

Among patients with drug-susceptible tuberculosis, 13 (30%) were classified as cured and 28 (65%) completed therapy, hence 41 (95%) achieved a successful treatment outcome according to the WHO 2021 outcome definitions (Supplementary Figure 1). Two patients (5%) died during treatment. Based on the TBnet outcome definitions, 33 (77%) were classified as cured, 2 (5%) died, and 8 patients (19%) had an undeclared treatment outcome.

**Figure 2.**
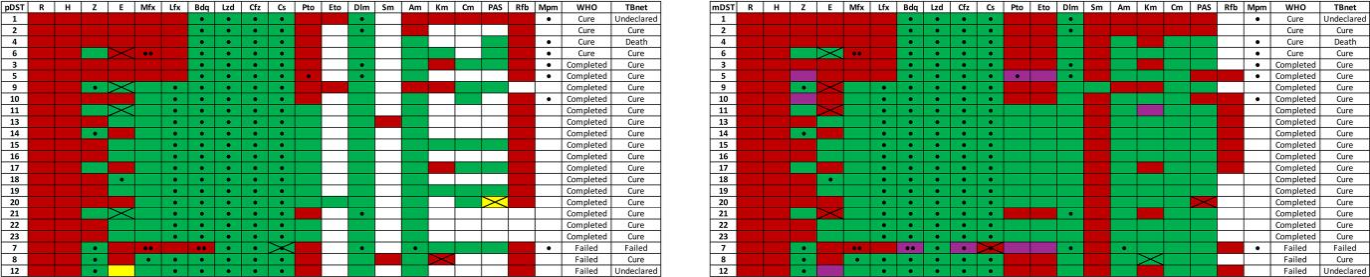
Individual molecular and phenotypic drug susceptibility test results in patients with multidrug-resistant or rifampicin-resistant (MDR/RR) tuberculosis. Each row represents one patient with MDR/RR tuberculosis with WHO and TBnet outcomes. *A*, Molecular drug susceptibility testing (mDST) by whole-genome sequencing (WGS) and *B*, Phenotypic drug susceptibility testing (pDST). Cell colors indicate results: green, susceptible; yellow, intermediate; red, resistant; purple, mutation with undefined resistance interpretation. A single dot within the cell indicates that the respective drug was administered as part of the treatment regimen, whereas two dots indicate that the drug was given at high dose. A diagonal cross indicates discordant results between mDST and pDST for the respective drug. Abbreviations: Am, Amikacin; Bdq, Bedaquiline; Cfz, Clofazimine; Cm, Capreomycin; Cs, Cycloserine; Dlm, Delamanid; E, Ethambutol; Eto, Ethionamide; H, Isoniazid; KmKanamycin; Lfx, Levofloxacin; Lzd, Linezolid; Mfx, Moxifloxacin; Mpm, Meropenem; PAS, Para-aminosalicylic acid; Pto, Prothionamide; R, Rifampicin; Rfb, Rifabutin; Sm, Streptomycin; Z, Pyrazinamide.

Among those with MDR/RR tuberculosis, 4 (17%) were classified as cured and 16 (70%) completed therapy, hence 20 (87%) achieved a successful treatment outcome according to the WHO 2021 outcome definitions (Figure 2). Three patients (13%) experienced treatment failure. One of these patients, who had a dissocial personality disorder, showed poor adherence to therapy and repeatedly had positive sputum cultures over several years. In another patient, protionamide and cycloserine had to be discontinued due to severe nausea, and pyrazinamide was stopped because of an allergic reaction. In the third patient, cycloserine was discontinued due to tongue discoloration, and linezolid was later discontinued because of progressive neuropathic symptoms. Based on the TBnet outcome definitions, 19 MDR/RR tuberculosis patients (83%) were classified as cured, 1 patient (4%) died, 1 (4%) experienced treatment failure, and 2 patients (9%) had an undeclared outcome.

### Regression Analysis of Factors Associated With Cure

In univariate Firth logistic regression, none of the investigated variables were significantly associated with cure according to the WHO definition. In the multivariate Firth logistic regression model, which included tuberculosis drug resistance group (MDR/RR vs drug-susceptible), age group (≤37 vs >37 years), sex, bilateral lung involvement, cavitary disease, extrapulmonary involvement, and BMI (<18.5 vs ≥18.5 kg/m^2^), no variable reached statistical significance. Full results of the univariate and multivariate analyses are presented in Supplementary Table 1.

### DST Results

**Figure 3.**
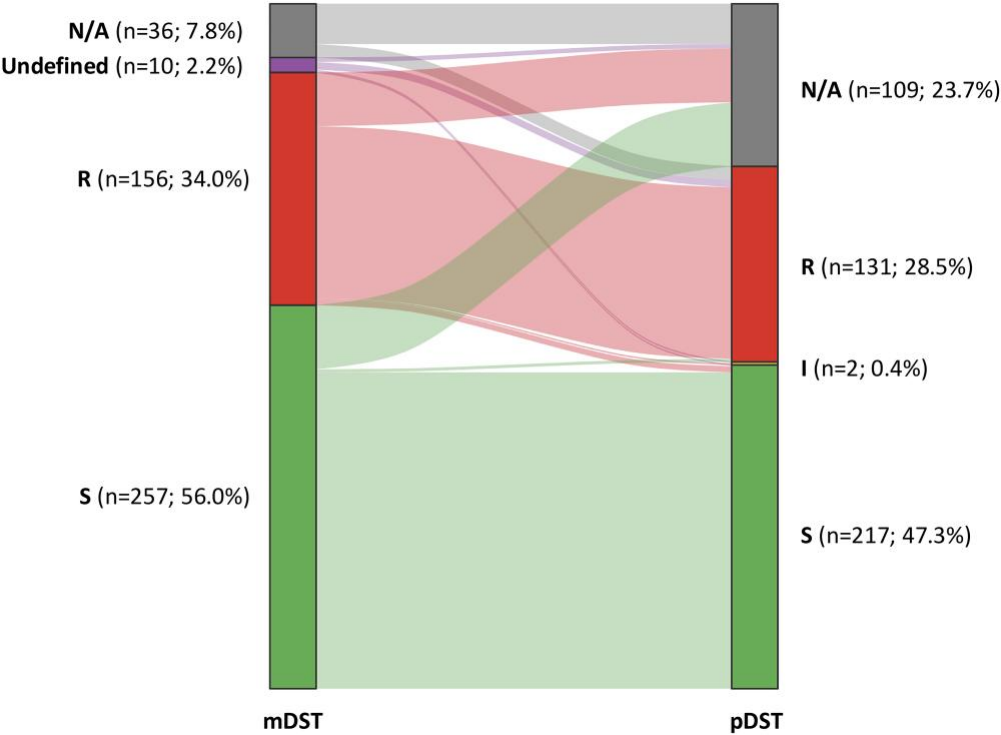
Comparison of molecular and phenotypic drug susceptibility testing in multidrug-resistant or rifampicin-resistant (MDR/RR) tuberculosis. Sankey diagram illustrating drug susceptibility testing results among patients with multidrug-resistant or rifampicin-resistant (MDR/RR) tuberculosis. Flows depict transitions from molecular drug susceptibility testing (mDST) to phenotypic drug susceptibility testing (pDST). Results are categorized as susceptible (S), resistant (R), intermediate (I), undefined resistance interpretation (undefined), or missing results (N/A). Numbers and percentages are shown for each category in the mDST and pDST columns.

Among patients with drug-susceptible tuberculosis isolate–drug combinations with results available from both mDST and pDST, no discordant results were observed for drugs classified as susceptible by both methods (Supplementary Figure 1). However, for a substantial proportion of isolate–drug combinations, results from one or both methods were not available because the respective drugs were not tested. Among patients with MDR/RR tuberculosis, 212 isolate–drug combinations were concordantly classified as susceptible and 115 as resistant, resulting in almost perfect agreement between pDST and mDST (Cohen's *κ* = 0.96) (Figures 2 and  3 and Supplementary Table 2). In addition, 27 isolate–drug combinations lacked results from both testing modalities. Only a small number of combinations represented genuine discrepancies between pDST and mDST. Specifically, 4 isolate–drug combinations were classified as resistant by mDST but susceptible by pDST, 2 as susceptible by mDST but resistant by pDST, and 1 as resistant by mDST with an intermediate pDST result. In total, this resulted in 7 true mismatches (Supplementary Table 3).

### Treatment Algorithms

**Figure 4.**
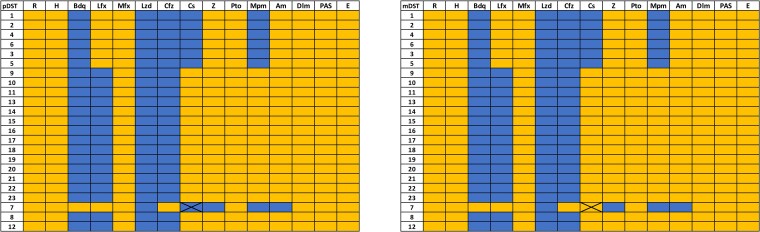
Algorithm-derived treatment regimens based on different methods of drug susceptibility testing for patients with multidrug-resistant or rifampicin-resistant (MDR/RR) tuberculosis. Each row represents one patient with MDR/RR tuberculosis. Regimens were based on respective results of molecular drug susceptibility testing (mDST) by whole-genome sequencing (WGS) (*A*) and phenotypic drug susceptibility testing (pDST) (*B*). Cell colors indicate results: blue means that the drug should be administered according to the algorithm, and yellow means that the drug should not be administered according to the algorithm. The differences in the resulting therapy regimes is highlighted by a diagonal cross. Meropenem was selected as per treatment algorithm, irrespective of the unavailability of DST. Abbreviations: Am, Amikacin; Bdq, Bedaquiline; Cfz, Clofazimine; Cs, Cycloserine; Dlm, Delamanid; E, Ethambutol; H, Isoniazid; Lfx, Levofloxacin; Lzd, Linezolid; Mfx, Moxifloxacin; Mpm, Meropenem; PAS, Para-aminosalicylic acid; Pto, Prothionamide; R, Rifampicin; Z, Pyrazinamide.

Using the standardized treatment algorithm, the regimens derived from pDST and mDST results were almost identical for all patients (Figure 4). In fact, for 65 of 66 patients, both approaches resulted in the same recommended combination of drugs. Only in a single case did the algorithm yield a different regimen: the pDST-based regimen suggested linezolid, cycloserine, pyrazinamide, meropenem, and amikacin, whereas the mDST-based algorithm omitted cycloserine due to a discordant cycloserine susceptibility result between mDST and pDST.

## DISCUSSION

In this cohort, patients with both drug-susceptible and MDR/RR tuberculosis achieved remarkably high treatment success rates under real-world conditions in a high-resource setting with access to comprehensive pDST and mDST. Remarkably, treatment outcomes among patients with MDR/RR tuberculosis were comparable to those with drug-susceptible disease. This contrasts sharply with global trends, where treatment success reaches approximately 88% for drug-susceptible tuberculosis but only around 71% for MDR/RR tuberculosis in the most recent WHO Global Tuberculosis Report—and was even as low as 60% during the years in which the patients in this study were treated [1]. This discrepancy may be attributable to the availability of extensive pDST and WGS-based mDST at the Research Center Borstel, which facilitated the design of optimized, individualized treatment regimens.

In light of the widespread lack of timely and reliable DST infrastructure, particularly for second-line tuberculosis drugs, in many high-burden countries, our findings strongly support the broader implementation of DST to improve patient outcomes and advance efforts against drug-resistant tuberculosis [16–$132#?>18]. The high concordance between WGS-based mDST and pDST observed in our analysis illustrates the robustness of molecular resistance prediction in routine clinical practice and adds to the growing body of evidence supporting the use of mDST as a frontline diagnostic tool. As resistance to essential drugs for the treatment of MDR/RR tuberculosis, such as fluoroquinolones and bedaquiline, continues to emerge [19–22], the rapid and accurate detection of resistance-conferring mutations becomes increasingly important. While WGS has proven highly effective in reference laboratories, its broad implementation in high-burden, resource-limited settings are constrained by costs, technical complexity, and the need for bioinformatics infrastructure. In this context, targeted next-generation sequencing (tNGS) has gained attention as a pragmatic alternative [18]. By focusing on a curated set of resistance-associated loci, tNGS can deliver results with shorter turnaround times, reduced sequencing and analytical requirements, and better feasibility for decentralized laboratory networks [23, 24].

Beyond diagnostic capacity, several additional factors likely contributed to the favorable outcomes in our study. Patients were treated in a specialized tuberculosis reference center with experienced multidisciplinary teams and close clinical monitoring, which likely improved adherence, facilitated early detection of drug toxicity, and enabled rapid treatment adjustments. This underscores that diagnostic innovation alone is insufficient and that it must be embedded within strong clinical and programmatic structures to achieve maximal patient benefit.

In addition to the high concordance between pDST and mDST, the treatment regimens derived from the standardized WHO-based algorithm were nearly identical regardless of whether pDST or mDST results served as input. For 65 of 66 patients, both approaches yielded the same recommended drug combination. Only in a single case did the algorithms diverge, where pDST suggested inclusion of cycloserine while the mDST-based algorithm omitted it due to a discordant susceptibility result. This finding reinforces that, in most cases, mDST provides sufficiently reliable information to guide individualized regimen design [4].

A further strength of this study is the use of 2 complementary treatment outcome frameworks. While the WHO 2021 definitions were used as the primary endpoint, reflecting programmatically relevant treatment outcomes, we additionally applied the TBnet criteria as a secondary analysis. The TBnet definition requires the absence of relapse within 1 year after treatment completion to classify a patient as cured and therefore provides a more stringent assessment of relapse-free cure. This complementary analysis supports the robustness of our findings.

To our knowledge, no studies have simultaneously integrated extensive pDST, WGS-based mDST, and both WHO 2021 and TBnet outcome definitions—making this cohort uniquely well-characterized and allowing for a particularly reliable interpretation of treatment effectiveness.

Several limitations should be acknowledged. First, patients in this cohort were treated before the introduction of newer shorter all-oral regimens for MDR/RR tuberculosis, such as the bedaquiline, pretomanid, linezolid ± moxifloxacin regimen and the 6- to 9-month regimens evaluated in the endTB and BEAT-TB trials [14, 25–27]. These regimens have demonstrated treatment success rates that exceed those of conventional 18-month therapies and approach outcomes typically seen in drug-susceptible tuberculosis. Nonetheless, our findings remain highly relevant: even with modern regimens, a substantial proportion of patients still require individualized therapy due to complex resistance patterns, drug toxicity, extensive extrapulmonary disease, or limited drug availability.

Second, the relatively small sample size limited our ability to assess additional predictors of treatment outcomes such as comorbidities, prior tuberculosis treatment, or immunosuppression and precluded meaningful comparisons between specific treatment regimens. In addition, the applied inclusion and exclusion criteria resulted in a highly selected cohort. We excluded patients with rifampicin resistance detected solely by molecular testing without phenotypic confirmation, those with ≥7 days of antituberculosis treatment prior to referral, and isolates demonstrating resistance to isoniazid, pyrazinamide, or ethambutol. Because phenotypic growth of *M. tuberculosis* is not always guaranteed and may be influenced by factors such as bacterial load, sample handling, or culture conditions, this approach may have excluded some true rifampicin-resistant cases without successful culture growth. While these criteria were chosen to ensure diagnostic certainty and cohort homogeneity, they may have introduced selection bias and may limit the generalizability of our findings to the broader population of patients with rifampicin-resistant or multidrug-resistant tuberculosis. Larger, multicenter cohorts including more heterogeneous patient populations will be essential to validate our findings and to determine whether similar outcomes can be achieved outside a highly specialized reference setting.

Finally, it is important to acknowledge that our cohort reflects a high-resource, low-incidence referral setting with access to comprehensive diagnostics, individualized treatment regimens, and close clinical monitoring. The encouraging outcomes observed in this context may therefore not be directly generalizable to lower-resource or high-burden settings. In such environments, limited access to advanced drug susceptibility testing, restricted availability of second-line drugs, higher rates of HIV co-infection, and constrained health-care infrastructure may substantially affect treatment implementation and outcomes. Caution is therefore warranted when extrapolating our findings beyond similarly specialized settings, and further studies in diverse health-care contexts are needed to assess external validity.

## CONCLUSIONS

In this real-world cohort from a high-resource setting, treatment outcomes for patients with MDR/RR tuberculosis were comparable to those with drug-susceptible disease. These findings highlight that optimized, individualized regimens guided by comprehensive pDST and mDST susceptibility testing can enable favorable outcomes even in drug-resistant tuberculosis. To improve global treatment success rates, expanding access to robust DST capacity should be prioritized—particularly in high-burden settings where such tools are most urgently needed.

## Supplementary Material

ofag349_Supplementary_Data

## Supplementary Data

Supplementary materials are available at *Open Forum Infectious Diseases* online. Consisting of data provided by the authors to benefit the reader, the posted materials are not copyedited and are the sole responsibility of the authors, so questions or comments should be addressed to the corresponding author.
