# Supplementary material for: Individualized Therapy Guided by Drug Susceptibility Testing for Multidrug-Resistant Tuberculosis
Source: Open Forum Infect Dis. 2026 Jun 18;13(6):ofag349. doi: 10.1093/ofid/ofag349 (PMC13308718; doi:10.1093/ofid/ofag349)
Supplement: ofag349_Supplementary_Data [file ofag349_supplementary_data.zip › Supplementary_Table_1_R2.docx]

|  | **Univariate analysis** | | **Multivariate analysis** | |
| --- | --- | --- | --- | --- |
| **Variable** | **OR (95% CI)** | **p-value** | **OR (95% CI)** | **p-value** |
| MDR/RR tuberculosis | 1.92 (0.6 - 7.08) | 0.28 | 2.84 (0.77 - 12.7) | 0.12 |
| Age > 37 years | 1.01 (0.34 - 2.99) | 0.99 | 1.59 (0.45 - 5.86) | 0.47 |
| Male gender | 3.02 (0.99 - 9.46) | 0.05 | 2.56 (0.83 - 8.33) | 0.10 |
| Bilateral disease | 1.38 (0.43 - 4.28) | 0.58 | 1.52 (0.43 - 5.3) | 0.51 |
| Cavitary disease | 0.91 (0.31 - 2.74) | 0.87 | 0.91 (0.26 - 3.13) | 0.88 |
| Extrapulmonary involvement | 0.16 (0 - 1.46) | 0.12 | 0.16 (0 - 1.67) | 0.14 |
| BMI <18.5 kg/m² | 1.13 (0.31 - 4.99) | 0.86 | 1.27 (0.29 - 6.52) | 0.75 |

**Supplementary Table 1.** Univariate and multivariate Firth logistic regression analyses for factors associated with cure

**Legend:** Abbreviations: BMI = Body mass index, CI = Confidence interval, MDR/RR = Multidrug- or rifampicin-resistant, OR = Odds ratio
