# Supplementary material for: Individualized Therapy Guided by Drug Susceptibility Testing for Multidrug-Resistant Tuberculosis
Source: Open Forum Infect Dis. 2026 Jun 18;13(6):ofag349. doi: 10.1093/ofid/ofag349 (PMC13308718; doi:10.1093/ofid/ofag349)
Supplement: ofag349_Supplementary_Data [file ofag349_supplementary_data.zip › Supplementary_Table_2_R2.docx]

**Supplementary Table 2.** Cross-classification of phenotypic drug susceptibility testing (pDST) and molecular drug susceptibility testing (mDST) results across all evaluated drugs among patients with multidrug-resistant tuberculosis (MDR-TB).

|  |  | **mDST** | | | |  |
| --- | --- | --- | --- | --- | --- | --- |
|  |  | Susceptible | Resistant | Unknown mutation | Not tested | **Total** |
| **pDST** | Susceptible | 212 | 4 | 1 | 0 | 217 |
|  | Resistant | 2 | 115 | 5 | 9 | 131 |
|  | Intermediate | 0 | 1 | 1 | 0 | 2 |
|  | Not tested | 43 | 36 | 3 | 27 | 109 |
|  | **Total** | 257 | 156 | 10 | 36 |  |

**Legend:** Abbreviations: pDST, phenotypic drug susceptibility testing; mDST, molecular drug susceptibility testing
