# Supplementary material for: Individualized Therapy Guided by Drug Susceptibility Testing for Multidrug-Resistant Tuberculosis
Source: Open Forum Infect Dis. 2026 Jun 18;13(6):ofag349. doi: 10.1093/ofid/ofag349 (PMC13308718; doi:10.1093/ofid/ofag349)
Supplement: ofag349_Supplementary_Data [file ofag349_supplementary_data.zip › Supplementary_Table_3_R1.docx]

**Supplementary Table 3.** Discordant results between whole genome sequencing (WGS)-based molecular drug susceptibility testing (mDST) and phenotypic drug susceptibility testing (pDST)

| **Patient** | **Drug** | **mDST** | **pDST** | **Drug in treatment regimen** | **Mutation** |
| --- | --- | --- | --- | --- | --- |
| **6** | E | S | R | no | embC R927R (likely benign); embA C76C, embB W332R (both benign); three mutations with unknown effect in ethambutol-region |
| **7** | CS | R | S | yes | *ald* del 9_16 |
| **8** | KM | S | R | no | *whiB7*_-117 t>c |
| **9** | E | R | S | no | embB G406A |
| **11** | E | R | S | no | *embA* -16 c>t |
| **20** | PAS | R | I | no | Rv2670c A5V, gene annotation: *ribD* -12g>a |
| **21** | E | R | S | no | embB G406S |

**Legend:** Abbreviations: E = Ethambutol; CS = Cycloserine; KM = Kanamycin; PAS = Para-aminosalicylic acid; mDST = Molecular drug susceptibility testing; pDST = phenotypic drug susceptibility testing; R = resistant; S = susceptible; I = intermediate.
