# Supplementary figures and images for: Individualized Therapy Guided by Drug Susceptibility Testing for Multidrug-Resistant Tuberculosis
Source: Open Forum Infect Dis. 2026 Jun 18;13(6):ofag349. doi: 10.1093/ofid/ofag349 (PMC13308718; doi:10.1093/ofid/ofag349)

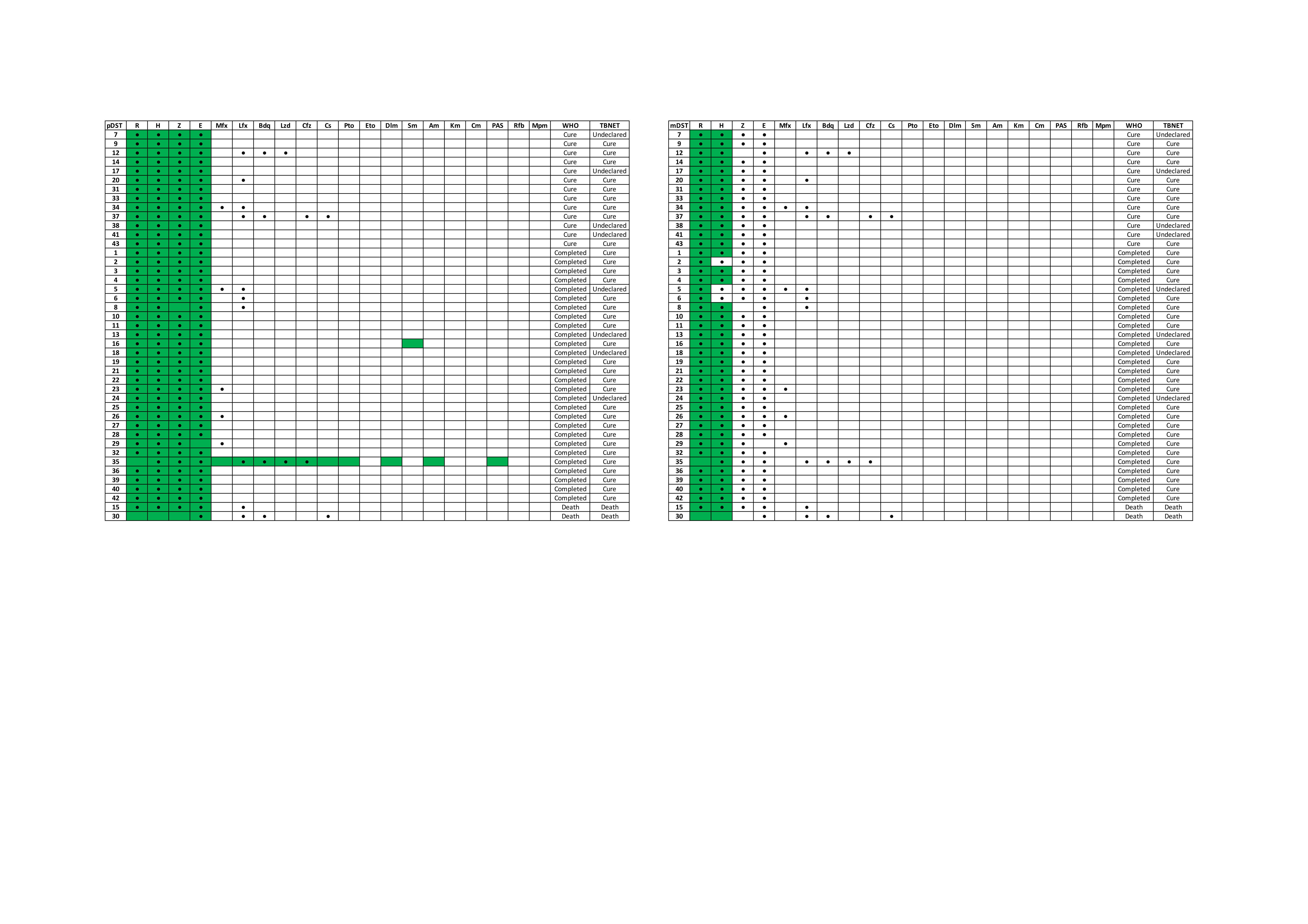

Supplement: ofag349_Supplementary_Data [file ofag349_supplementary_data.zip › Supplemental_Figure_1.png]
